# In Vitro Evaluation of Acellular Collagen Matrices Derived from Porcine Pericardium: Influence of the Sterilization Method on Its Biological Properties

**DOI:** 10.3390/ma14216255

**Published:** 2021-10-21

**Authors:** Rone Aparecido De Grandis, Larissa Natiele Miotto, Luis Eduardo Genaro, Larissa Migliatti Polli, Ana Maria de Guzzi Plepis, Fabiana Tessari Rodrigues, Virginia da Conceição Amaro Martins, Leonardo Pereira Franchi, Raquel Mantuaneli Scarel-Caminaga, Ticiana Sidorenko de Oliveira Capote

**Affiliations:** 1 Faculty of Pharmaceutical Sciences-FCFar, Paulista State University, Araraquara 14801-903, SP, Brazil; degrandis.rone@fcfar.unesp.br (R.A.D.G.); lari.mpolli@gmail.com (L.M.P.); 2 Department of Morphology, Genetics, Orthodontic and Pediatric Dentistry, Araraquara School of Dentistry, Paulista State University, Araraquara 14801-903, SP, Brazil; larissanmiotto@foar.unesp.br (L.N.M.); luis-genaro@outlook.com (L.E.G.); raquel@foar.unesp.br (R.M.S.-C.); 3 Department of Chemistry and Molecular Physics, Institute of Chemistry of São Carlos, University of São Paulo, Sao Carlos 13566-590, SP, Brazil; amplepis@iqsc.usp.br (A.M.d.G.P.); fabianatessari@gmail.com (F.T.R.); virginia@iqsc.usp.br (V.d.C.A.M.); 4 Department of Genetics, Faculty of Medicine of Ribeirão Preto, University of São Paulo, Sao Carlos 13566-590, SP, Brazil; leonardofranchi@yahoo.com.br

**Keywords:** porcine pericardium, tissue regeneration, collagen matrices

## Abstract

The aim of this study were characterize acellular collagen matrices derived from porcine pericardium (PP) and to evaluate their properties after sterilization by ethylene oxide and gamma ray. PP matrices were subjected to alkaline hydrolysis (AH), and samples were characterized for biological stability, membrane thickness measurements, differential scanning calorimetry (DSC) and scanning electron microscopy (SEM). Subsequently, the matrices were frozen, lyophilized and sterilized by ethylene oxide or gamma radiation. For in vitro assays, CHO-K1 cell culture was used and evaluated for cytotoxicity, clonogenic survival assay, genotoxicity and mutagenicity. Analysis of variance (ANOVA) was used, followed by Dunnett’s post-test, with a significance level of 5%. After AH, there was no significant change in matrix thickness. The relative biodegradability of the material after implantation was observed. Morphology and dimensions had small changes after AH. As for cell viability, none of the tested matrices showed a statistically significant difference (*p* > 0.05; Dunnett) regardless of the sterilization method. Furthermore, it was found that PP matrices did not interfere with the proliferation capacity of CHO-K1 cells (*p* > 0.05; Dunnett). As for genotoxicity, when sterilized with ethylene oxide (NP, P12 and P24), it showed genotoxic potential, but it was not genotoxic when sterilized by gamma radiation. No mutagenic effects were observed in either group. PP-derived collagen matrices hydrolyzed at different times were not cytotoxic. It is concluded that the best method of sterilization is through gamma radiation, since no significant changes were observed in the properties of the PP matrices.

## 1. Introduction

Collagen of animal origin is recognized as one of the most useful biomaterials available and is widely used in tissue engineering, cosmetic surgeries and drug delivery systems [1]. Due to the properties of repair, biodegradability and biocompatibility, the collagen matrices are materials of great utility, mainly for the medical and dental area. In dentistry, collagen-based matrices can be used in periodontal reconstruction in a process known as guided tissue regeneration. In these cases, it is interesting that the materials are resorbable to eliminate a second surgery [2].

Resorbable collagen matrices can be obtained from a variety of materials, such as porcine pericardium [2]. The pericardium is one of the three tunics that cover the heart: the innermost tunic is called the endocardium, the middle is the myocardium, and the outermost is the pericardium. One of the great advantages of using PP in the development of biomaterials is the high content of type I collagen, with approximately 47% in these tissues. Collagen has a large amount of reactive groups, such as amines (NH_2_), carboxylic acids (COOH) and alcoholic hydroxyls (OH), which enable chemical changes in the tissue, mainly through crosslinking reactions [3,4,5,6,7,8]. Plepis et al. [9] performed the preparation of acellular matrices of type I collagen by alkaline hydrolysis of porcine pericardium at different times and verified that the collagen fibers were preserved and the cells were removed after alkaline hydrolysis, showing that the matrix became acellular. In this way, the use of these matrices becomes quite interesting, since besides the possibility of chemical modifications, they have advantages such as low cost, great availability and being easy to obtain.

Before using these matrices, the sterilization of these materials must be carried out to avoid contamination of the region where the biomaterial is implanted. However, common methods of sterilization (autoclaving, irradiation, ethylene oxide) may induce changes that may affect mechanical resistance or performance [10], and it is necessary to find ways to minimize these effects. Ethylene oxide is a colorless and flammable gas which is widely used for the sterilization of medical equipment, but there are reports of negative effects on some materials, such as the production of a toxic chemical residue, ethylene chlorohydrin, requiring a period for aeration of the material for its elimination [11,12,13]. The same response, however, is less frequent in groups sterilized by gamma radiation, since it is free of residues and it is a more advantageous method [11].

Depending on the type of biomaterial, it is necessary to carry out several standardized tests before its use (ISO 10993-12) [14]. Chemical, physical and biological products can interact with genetic material, resulting in mutations that are associated with genomic instability and cancer [15]. In vitro methods have a shorter runtime, provide meaningful data more easily [16] and provide important tools to improve extrapolation in human in vivo assays [17].

As porcine pericardium matrices can be used for biological grafts and stay in contact with the patient for a period longer than fifteen days, cytotoxicity and genotoxicity tests are required. Furthermore, as the sterilization method may also interfere in these matrices, it is important to evaluate if it has an effect. Based on that, the aim of this study was to characterize acellular collagen matrices derived from porcine pericardium (without and with 8, 12 and 24 h of alkaline hydrolysis) and to evaluate cytotoxic, genotoxic and mutagenic effects after sterilization by ethylene oxide.

## 2. Materials and Methods

### 2.1. Preparation of Porcine Pericardium Matrices

Porcine pericardium (PP) was obtained from Braile Biomedica S/A, São José do Rio Preto-SP, Brazil, and it was subjected to alkaline hydrolysis (AH). For AH, PP was immersed for 8 (P8), 12 (P12) and 24 h (P24) at 25 °C in an alkaline solution containing salts of K^+^, Na^+^ and Ca^++^ for 6 h. Excess salts were removed by rinses in 3% (w/w) boric acid solution, deionized water, followed by 0.3% (w/w) EDTA solution, pH 11 and finally equilibrated in 0.13 mol L^−1^ phosphate buffer, pH 7.4 [9]. The pericardium matrices were frozen, lyophilized and sterilized by ethylene oxide or by gamma radiation (20 kGy).

### 2.2. PP Matrices Characterization

The characterization was made by biological stability (collagenase), measurements of membrane thickness, differential scanning calorimetry (DSC) and scanning electron microscopy (SEM).

After the samples were frozen and lyophilized to constant weight, 3 discs of 1 cm diameter for each sample were obtained. Collagenase solution (10 U mg^−1^ collagen) in Tris-HCl buffer pH 7.4 with an activity of 30 U mL^−1^ was added to the samples and then incubated at a temperature of 37 °C for 2 h. After this period, the samples were washed with deionized water, frozen, lyophilized to constant weight and weighed.

The percentage of degraded collagen mass (% degradation) was determined from the difference of mass before collagen (initial M) and after the enzymatic degradation (final M) and calculated as % degradation = (initial M − final M) × 100/initial M, with the results being an average of three independent determinations.

The denaturation temperature (dT) of the matrices was determined using a Differential Scanning Calorimeter (DSC equipment model, TA Instruments, New Castle, DE, USA), calibrated with an indium standard. The samples were equilibrated for 24 h with phosphate buffer and a pH of 7.4, and measurements were performed in a hermetically sealed atmosphere of synthetic air (80 mL min^−1^) at a heating rate of 10 °C min^−1^ and a temperature range of 5 to 120 °C. The masses of the samples were approximately 10 mg, and dT values were obtained from the inflection point of the DSC curve.

The SEM analyses were performed on LEO 440 equipment (LEO Electron Microscopy Ltd., Waltham, MA, USA) operating at 20 keV beam electrons. The samples were coated with 20 nm palladium-gold alloy in a metallizer Balsers SDS 050.

### 2.3. Cell Culture Experiments

CHO-K1 cells were cultured in 1:1 Ham-F10 + D-MEM medium (Sigma-Aldrich, San Luis, MO, USA) supplemented with 10% fetal bovine serum (Cultilab, Campinas, Brazil) and antibiotics solution (penicillin/streptomycin/kanamycin/ciprofloxacin) in 25 cm^2^ culture flasks at 37 °C, 5% CO_2_. Cells were used between the third and eighth passages.

### 2.4. Cytotoxic Assays

#### 2.4.1. XTT Assay

CHO-K1 cells (2 × 10^4^) were seeded in 24-well plates and exposed for 24 h to native pericardium (NP), P8, P12 and P24 sterilized by ethylene oxide (in duplicate). Negative controls (NC) were wells without any matrix (untreated controls), while positive controls (PC) were treated with 0.001 M hydrogen peroxide (Merck, Darmstadt, Germany) during 10 min in the dark room. After 24 h of incubation, the cultures were washed with PBS solution, and 500 µL of DMEM without phenol red (Sigma) were immediately added, followed by the addition of 60 µL of the XTT/electron solution (50:1) (Kit-XTT, Roche Molecular Biochemicals, Mannhein, Germany); this was incubated for 2 h. Next, the supernatant was transferred to a 96-well plate and a colorimetric reading was taken in a spectrophotometer (Ultrospec™ 2100 pro UV/Visible Spectrophotometer, Fisher Scientific International, Hampton, VA, USA). The result of the absorbance measured at 492 and 690 nm is directly proportional to the number of viable cells in each treatment after 24 h of exposure.

#### 2.4.2. Clonogenic Survival Assay

Procedures were followed as previously described [18]. Briefly, CHO-K1 cells (5 × 10^4^) were exposed for 24 h to NP, P8, P12 and P24 sterilized by ethylene oxide in 24-well plates; negative controls (NC) were wells without matrices, while positive controls (PC) were treated with doxorubicin (0.3 μg·mL^−1^) for 4 h (all experiments were carried out in duplicate). After exposure, the cultures were washed with PBS, and fresh medium was added. Exponentially growing cells were seeded at a number of 150 cells per 25 cm^2^ flask, in duplicate for each treatment. The flasks were incubated at 37 °C, 5% CO_2_ for 7 days without culture medium change. The colonies that formed were fixed with methanol:acetic acid:water (1:1:8 *v*/*v*/*v*) and stained with 5% Giemsa. The colonies were counted, and the cell-surviving fraction was calculated as the percent of colonies in treated flasks relative to untreated controls (NC).

### 2.5. Genotoxic Assay

#### Comet Assay

For detection of DNA strand breaks, the single cell gel electrophoresis or comet assay was used in the alkaline version, based on the method of Singh et al. [19]. The treatments and cell culture were executed similarly to the clonogenic survival assay. After exposure to NP, P8, P12 and P24 previously sterilized by ethylene oxide or by gamma radiation, the cells were washed with PBS and released by trypsin. The cell suspension was centrifuged at 500 rpm for 5 min at 4 °C. The pellet was resuspended in 200 μL of 0.5% (w/v) low melting point agarose (Sigma-Aldrich) and the mixture was spread onto two microscope slides (Waldemar Knittel Glasbearbeitungs GmbH, Wildhagen, Germany) pre-coated with 1.5% (w/v) normal melting point agarose (Gibco, Dun Laoghaire, Ireland). Cover slides were placed over the gel. When the gels had solidified, the cover slides were gently removed and the slides were immersed in cold (4 °C) lysis solution (1% Triton X-100, 10% DMSO, 2.5 mmol·L^−1^ NaCl, 100 mmol·L^−1^ Na_2_EDTA, 100 mmol·L^−1^ Tris, pH = 10) for 24 h. Immediately after this step, slides were placed in a horizontal electrophoresis unit containing freshly prepared electrophoresis buffer (1 mmol·L^−1^ Na_2_EDTA, 300 mmol·L^−1^ NaOH, pH > 13). The DNA was allowed to unwind for 20 min, and subsequently, electrophoresis was performed at 25 V, 300 mA for 20 min. Afterwards, the slides were gently immersed in neutralization buffer (0.4 mol·L^−1^ Tris–HCl, pH = 7.5) for 15 min and then fixed with ethanol. Duplicate slides were prepared and stained with SYBR Green 2X (Invitrogen, Eugene, OR, USA), and 50 cells were screened per sample with a fluorescent microscope (ZEISS^®^, Jena, Thuringia, Germany) equipped with an excitation filter of 515–560 nm, a barrier filter of 590 nm and a 40× objective. The level of DNA damage was assessed by an image analysis system (TriTek CometScore^®^ 1.5, 2006, Sumerduck, VA, USA), and the DNA percent in the tail was obtained for each treatment.

### 2.6. Mutagenic Assay

#### Cytokinesis-Blocked Micronucleus (CBMN) Assay

The CBMN assay was performed according to Fenech [20,21]. CHO-K1 cells were seeded in 24-well plates at a density 5 × 10^4^ cells/well. After 24 h of seeding, cells were exposed for 24 h to the NP, P8, P12 and P24 matrices previously sterilized by ethylene oxide or by gamma radiation; negative controls (NC) were wells without any matrices, and positive controls (PC) were treated with doxorubicin (0.3 μg·mL^−1^) for 4 h (all experiments were carried out in duplicate). Cytochalasin-B (CytB) (Sigma) was added to the CHO-K1 cultures at a final concentration of 5 μg·mL^−1^ and left for 20 h. After the treatments, the cultures were washed with PBS, trypsinized and centrifuged for 7 min at 1500 rpm. The pellet was then resuspended in cold hypotonic solution (0.3% KCl w/v) for 3 min. The cells were fixed with methanol:glacial acetic acid (3:1, *v*/*v*) and formaldehyde. The cell suspensions were dripped on a slide with a film of distilled water at 4 °C. The slides were stained with 5% Giemsa solution diluted in phosphate buffer for 7 min, washed with distilled water, air dried and examined by light microscopy (400× magnification). One thousand (1000) cells were scored to evaluate the percentage of mono-, bi-, tri- and tetra-nucleated cells. The nuclear division index (NDI) was calculated according to the formula: [NDI = M1 + 2(M2) + 3(M3) + 4 (M4)/N], where M1–M4 represents the number of cells with 1–4 nuclei, respectively, and N is the total number of scored cells. Micronuclei (MNi) and Nucleoplasmic Bridges (NPBi) were scored in 1000 binucleated cells. MNi and NPBi are a biomarker of DNA damage and instability. The criteria for identifying micronucleus (MN) were based on Fenech [21].

### 2.7. Statistical Analysis

At least three independent experiments were conducted for each parameter analyzed. The experimental results are expressed as mean and standard error. The Shapiro−Wilk test was utilized to assess the normality of the data, and for homogeneity, the Levene test was utilized. In view of the results, parametric tests were utilized. The results were subjected to ANOVA (assuming *p* < 0.05) followed by Dunnett’s post-test for comparison with the negative control. The BioEstat statistical package (Version 5, UFPA, Guamá, Pará, Brazil) was used to perform the tests.

## 3. Results

All matrices were equilibrated in phosphate buffer (PB) and lyophilized. In all cases, they presented white coloration, heterogeneous texture and fine thickness. The thickness values are described in Table 1, showing that after the alkaline hydrolysis, there was no significant change in the thickness of the matrices.

Biological stability assays by collagenase were performed as a relative indication of the biodegradability of the material after implantation. The percentages of enzymatic degradation are described in Table 1.

These results show that after HA, there was an increase in enzyme degradation, which was greater with increasing hydrolysis time. This is because alkaline hydrolysis makes matrices more susceptible to enzymatic degradation.

In the DSC analysis, the denaturation temperature of the collagen present in the matrices was determined. This transition is related to the collagen → gelatin transition, showing that collagen was not denatured during alkaline hydrolysis, since gelatin does not present a thermal transition in the studied temperature range.

Comparing the dT values with the percentage of enzymatic degradation values, it appears that the lower the dT, the greater the enzymatic degradation percentage. These results occur because alkaline hydrolysis generates an increase in the number of negative charges on the collagen molecule as a function of the hydrolysis time, changing the charge distribution of the collagen molecules causing an electrostatic repulsion between them, making the matrix more susceptible to enzymatic degradation and denaturation.

**Figure 1:**
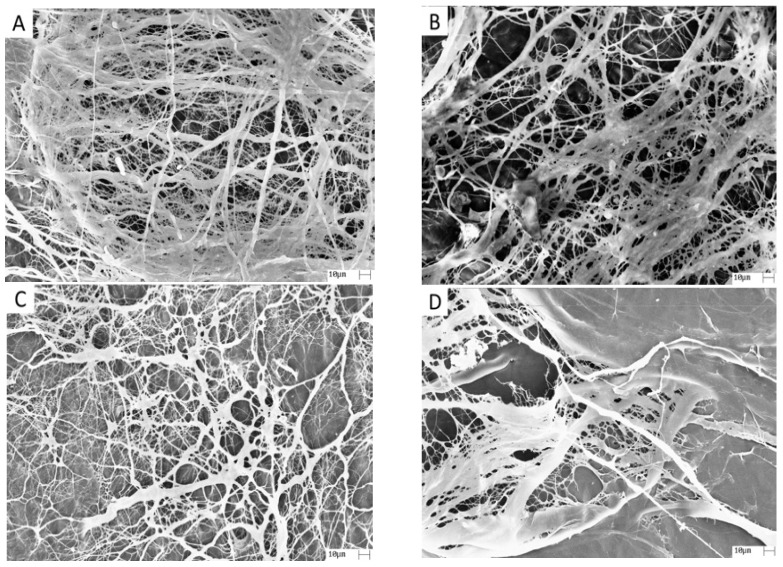
Photomicrographs of porcine pericardium matrices: (**A**) Group NP; (**B**) Group P8; (**C**) Group P12; and (**D**) Group P24. 1000× magnification.

Pericardium has a fibrous surface (outer surface) and a smooth surface (internal surface), and SEM was performed on the fibrous surface of the matrix (Figure 1). Figure 1A shows the fibrous surface structure of the NP matrix with the collagen fibers in a random orientation with interstices of varying sizes and shapes. The morphology and dimensions had small changes after alkaline hydrolysis (Figure 1B–D), showing that the hydrolysis time had little effect on the morphology of the fibrous surface of the matrices.

**Figure 2:**
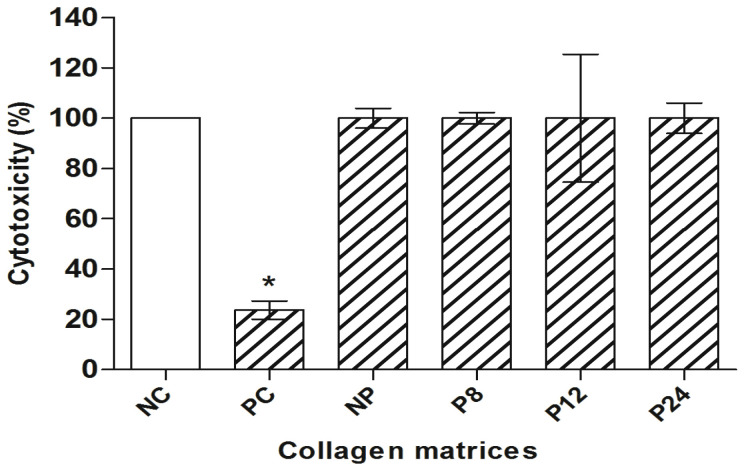
XTT assay in CHO-K1 (cell viability). NC: negative control; PC: positive control. NC represents 100% cell viability. Columns = mean of cell viability (%); bars = standard error. * = *p* < 0.01 compared to NC, Dunnett’s test.

In the present study, the cytotoxicity analysis was performed by the XTT assay. The mean and standard error of cell viability obtained by the XTT assay can be seen in Figure 2.

Considering the cell viability obtained from the NC as a reference (Dunnett’s), none of the tested matrices presented a statistically significant difference (*p* > 0.05, Dunnett’s).

**Figure 3:**
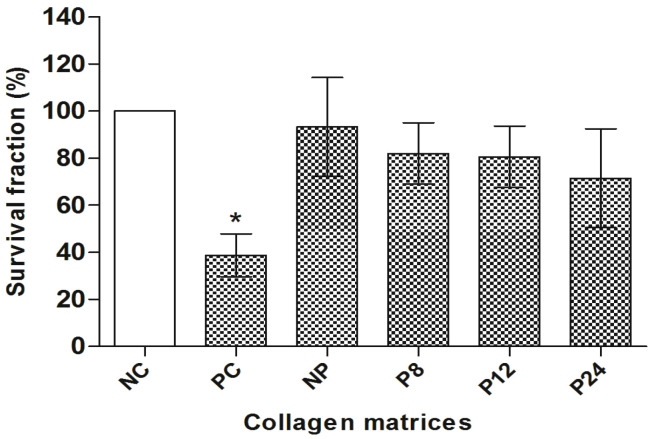
Clonogenic survival assay in CHO-K1. NC: negative control; PC: positive control. NC represents 100% survival fraction. Columns = mean of survival fraction (%); bars = standard error. * = *p* < 0.01 compared to NC, Dunnett’s test.

Figure 3 shows the survival fraction obtained by the clonogenic survival assay. It was verified that the porcine pericardium matrices did not interfere in the proliferation capacity of the CHO-K1 cells, since no significant difference was observed between them and NC (*p* > 0.05, Dunnett’s).

**Figure 4:**
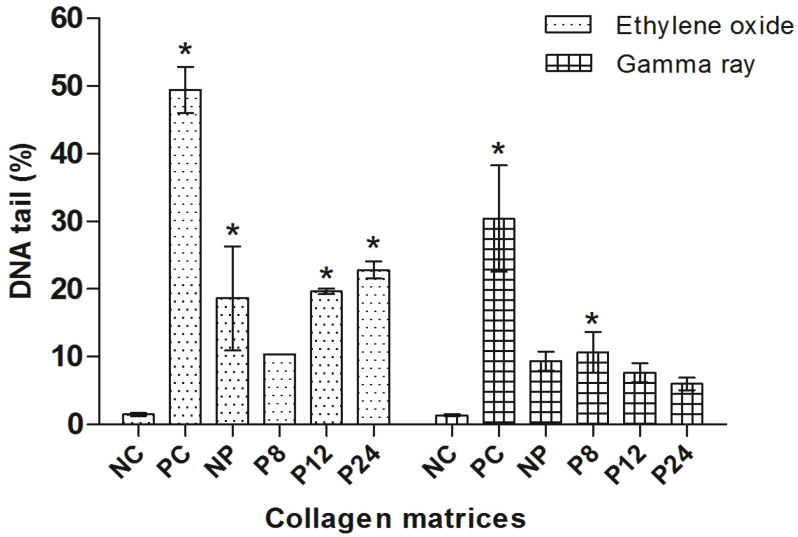
Comet assay in CHO-K1. NC: negative control; PC: positive control. Columns = mean of percentage of DNA in tail; bars = standard error. * = *p* < 0.01 compared to NC, Dunnett’s test.

The comet assay was carried out with the objective of ascertaining the genotoxic profile of the porcine pericardium matrices and comparing its effect depending on the type of sterilization method. These data are observed in Figure 4.

The analysis of variance (ANOVA) showed a statistically significant difference between the PC and the NC as well as in relation to all the treatments of the matrices sterilized with ethylene oxide. NP, P12, P24 and PC showed a statistically significant difference when compared to the NC (*p* < 0.01, Dunnett’s), indicating a genotoxic response. Only the treatment with the P8 group showed no significant difference when compared to the NC.

For the group of the matrices sterilized by gamma radiation, only PC and P8 presented a statistically significant difference compared to NC (*p* < 0.05, Dunnett’s); possibly, there was no difference due to the shorter time used for sterilization.

The micronucleus test is a widely used test for the evaluation of the mutagenic potential. The frequency of micronuclei (MNi) and nucleoplasmic bridges (NPBi) in binucleate cells were evaluated, which demonstrate additional chromosomal damage information for sterilized matrices by ethylene oxide and gamma ray radiation (Table 2).

For both groups of matrices sterilized with ethylene oxide and gamma radiation, a high number of micronuclei and nucleoplasmic bridges were observed only in the PC, which was also expected and which was statistically different from NC (*p* < 0.05, Dunnett’s). No significant differences were observed between the different treatments (NP, P8, P12 and P24) and in relation to NC.

## 4. Discussion

Porcine pericardial tissue is widely used as a biomaterial for tissue repair [4,5,6,7,8]. These types of xenografts, when acellular, are suitable for transplant purposes as they preserve the extracellular matrix, allowing the restoration of tissue viability and function through recellularization [5].

In the present study, we observed that after alkaline hydrolysis, there was an increase in enzymatic degradation, which was greater with increasing hydrolysis time (Table 1). A viable explanation is that alkaline hydrolysis makes matrices more susceptible to enzymatic degradation [22]. Through DSC analysis, the denaturation temperature of the collagen present in the matrices was determined. This transition is related to the collagen → gelatin transition, showing that collagen was not denatured during alkaline hydrolysis, since gelatin does not present a thermal transition in the studied temperature range [23].

Comparing the values of denaturation temperature (dT) with the percentage values of enzymatic degradation, it appears that the lower the dT, the greater the percentage of enzymatic degradation. These results occur because alkaline hydrolysis generates an increase in the number of negative charges on the collagen molecule as a function of the hydrolysis time [3], changing the charge distribution of the collagen molecules causing an electrostatic repulsion between them, making the matrix more susceptible to enzymatic degradation and denaturation.

Sterilization is necessary before the implantation of any biomedical device [24,25]. The ideal sterilization method should avoid significant changes in device properties [26]. Some sterilization methodologies range from the use of heat and pressure, ionizing radiation (ultraviolet (UV), X-ray, gamma irradiation and E-beam), chemical sterilants (ethylene oxide, hydrogen peroxide and peracetic acid) and, recently, the use of supercritical carbon dioxide and ionized gas plasma [24]. However, these sterilization procedures induce damage, altering their biomechanical and physiological properties and cell attachment [5,24,27].

Ethylene oxide is very useful as a sterilizing agent, which proceeds through the direct alkylation of the cellular constituents of the organisms, leading to denaturation. This method has advantages such as effectiveness at low temperatures, high penetration and compatibility with a wide range of materials. However, it is flammable and explosive, can produce toxic residues and reacts with functional groups such as amines [28].

Horakova et al. (2018) used a polyester biomaterial and observed that the fibroblast proliferation rate in samples sterilized with ethylene oxide was slower than in samples treated with ethanol; however, it did not show cytotoxicity to the cells.

It was possible to observe that the hydrolyzed porcine pericardium-derived collagen matrices at different times were not cytotoxic (Figure 2). Results that corroborate the study by Plepis et al. [9], when using porcine pericardium matrices, observed that they were not cytotoxic in an in vitro study and after implantation in subcutaneous Wistar rats, the matrices presented good biocompatibility and low inflammatory response; however, the sterilization method was not mentioned in this study.

Another method of sterilization is by gamma-ray irradiation, which can easily and uniformly reach all parts of the object to be sterilized; sterilization can be carried out with different doses for various materials in various physical states. However, irradiation with high-energy gamma rays can cause several changes in material properties, such as crosslinking and degradation [29].

When evaluating the genotoxicity of the matrices, we observed that the ethylene oxide sterilization method increased the percentage of DNA in the nucleoid tail (Table 2). Correlating the genotoxicity results found by comet assay, matrices irradiated with gamma rays showed low genotoxic potential. From the evaluations carried out, it was also verified that the matrices sterilized by ethylene oxide showed greater induction of DNA damage, showing genotoxic behavior. Thus, sterilization by gamma radiation and ethylene oxide are possible methods of sterilizing biomaterials made from type I collagen, but sterilization by gamma rays is preferable from a genotoxic point of view.

According to the results obtained, the swine pericardium matrices were not mutagenic, regardless of the sterilization method. Cândido-Bacani et al. [30] demonstrated that mutagenic effects may be related to the dose and time of exposure to the agent under investigation. A tendency towards an increase in the frequency of micronuclei could be verified with the increase in the hydrolysis time performed in porcine pericardium matrices. This result demonstrates that matrices treated with a shorter hydrolysis time may have lower mutagenicity.

As the induction of DNA damage must respect the dosage and exposure time of the material or substance tested, it was found that even at different times of hydrolysis and in different repetitions of the experiment, the porcine pericardium collagen matrices did not show mutagenic potential in both sterilization methods. Therefore, it can be inferred that the genotoxic response observed by the comet assay did not form mutations, according to the micronucleus assay. Thus, both sterilizations are possible to be carried out safely from a mutagenic point of view.

## 5. Conclusions

With the results obtained in this work, it can be concluded that collagen matrices derived from porcine pericardium hydrolyzed at different times were not cytotoxic in CHO-K1 cells by Clone Survival and XTT assays. It can also be concluded from the analysis of the genotoxicity results that the same matrices sterilized with ethylene oxide (NP, P12 and P24) presented genotoxic potential evaluated by the comet assay, while the group of membranes sterilized by gamma radiation did not demonstrate this effect at the same hydrolysis times (NP, P12 and P24). No mutagenic effects, however, were observed in either group. Thus, these matrices are a viable procedure for use in the production of biomaterials for tissue engineering.

## Figures and Tables

**Table 1:** Thickness as a function of the alkaline hydrolysis time, enzymatic degradation percentages and denaturation temperature (dT) obtained by DSC of pericardium matrices.

Matrix	Thickness (mm)	% Degradation *	dT (°C)
NP	0.19 ± 0.07	22.1 ± 1.1	71.2
P8	0.18 ± 0.07	32.5 ± 0.9	64.0
P12	0.21 ± 0.07	41.4 ± 0.8	63.5
P24	0.23 ± 0.11	69.7 ± 1.2	58.5

* mean values of 3 determinations.

**Table 2:** CBMN assay in CHO-K1. Mean and standard error of nuclear division index (NDI), frequency of micronuclei (MN) and frequency of nucleoplasmic bridges (NB) of collagen membranes sterilized by ethylene oxide (EO) and gamma ray (GR). * = *p* < 0.05 (Tukey and Dunnett’s).

Treatment	NDI (EO)	NDI (GR)	MN (EO)	MN (GR)	NPBi (EO)	NPBi (GR)
NC	1.802 ± 0.2	1.801 ± 0.0	3.0 ± 1.0	1.0 ± 1.7	1.3 ± 1.5	0.0 ± 0.6
PC	1.882 ± 0.1	1.684 ± 0.0	353.7 ± 67.4 *	32.0 ± 12.5 *	12.0 ± 3.6 *	8.0 ± 1.5 *
NP	1.855 ± 0.1	1.849 ± 0.1	9.3 ± 2.9	2.0 ± 2.8	3.0 ± 1.0	2.0 ± 2.1
P8	1.817 ± 0.1	1.831± 0.1	7.0 ± 1.7	5.0 ± 2.5	2.0 ± 1.0	1.0 ± 1.7
P12	1.882 ± 0.1	1.858 ± 0.1	8.0 ± 2.6	5.0 ± 3.6	2.0 ± 1.0	1.0 ± 0.6
P24	1.834 ± 0.1	1.871 ± 0.1	10.0 ± 2.0	6.0 ± 4.0	4.0 ± 1.7	2.0 ± 0.6

## Data Availability

Not applicable.
